# Modeling Autism Spectrum Disorders with Induced Pluripotent Stem Cell-Derived Brain Organoids

**DOI:** 10.3390/biom13020260

**Published:** 2023-01-30

**Authors:** John Lenon de Souza Santos, Cecília de Almeida Araújo, Clarissa Araújo Gurgel Rocha, Zaquer Suzana Munhoz Costa-Ferro, Bruno Solano de Freitas Souza

**Affiliations:** 1 Gonçalo Moniz Institute, Oswaldo Cruz Foundation (FIOCRUZ), Salvador 40296-710, Brazil; 2 Center for Biotechnology and Cell Therapy, São Rafael Hospital, Salvador 41253-190, Brazil; 3 D’Or Institute for Research and Education (IDOR), Salvador 41253-190, Brazil

**Keywords:** brain organoids, autism spectrum disorder, pluripotent stem cell

## Abstract

Autism spectrum disorders (ASD) are a group of complex neurodevelopmental disorders that affect communication and social interactions and present with restricted interests and repetitive behavior patterns. The susceptibility to ASD is strongly influenced by genetic/heritable factors; however, there is still a large gap in understanding the cellular and molecular mechanisms underlying the neurobiology of ASD. Significant progress has been made in identifying ASD risk genes and the possible convergent pathways regulated by these gene networks during development. The breakthrough of cellular reprogramming technology has allowed the generation of induced pluripotent stem cells (iPSCs) from individuals with syndromic and idiopathic ASD, providing patient-specific cell models for mechanistic studies. In the past decade, protocols for developing brain organoids from these cells have been established, leading to significant advances in the in vitro reproducibility of the early steps of human brain development. Here, we reviewed the most relevant literature regarding the application of brain organoids to the study of ASD, providing the current state of the art, and discussing the impact of such models on the field, limitations, and opportunities for future development.

## 1. Introduction

Autism spectrum disorder (ASD) is characterized by persistent difficulties in communication and social interactions. In addition, restricted and repetitive patterns of behaviors, interests, and activities are observed [1]. According to recent epidemiological data, the overall ASD prevalence in the United States is 23.0 per 1000 (1:44) among children aged eight years, and it is four times more common in boys than in girls [2]. Individuals with ASD may present with a variety of symptoms and levels of severity, ranging from mild to severe cases, requiring lifelong support and care [1,3].

ASD can be classified as (i) syndromic, which corresponds to rare and severe conditions accompanied by pathognomonic history or clinical findings (i.e., Fragile X syndrome, Phelan-McDermid syndrome, and others), or (ii) idiopathic, which comprises most cases. Approximately 80% of ASD cases do not have a clear etiology; however, diverse mutations, genetic variants, and environmental and epigenetic factors can interact and contribute as risk factors for the development of the disorder [4,5].

Despite being numerous, ASD risk genes converge to a limited number of biological processes involved in critical steps of neurodevelopment [6]. Some mutations may affect excitatory or inhibitory neurons, different neurotransmitter systems, and specific neuronal cell populations in the cerebral cortex or cerebellum [7,8]. Increasing knowledge of the key mechanisms involved in the neurobiology of ASD is critical for identifying novel potential therapeutic targets. To accomplish this, the impact of genetic variation at the cellular and molecular levels during neurodevelopment must be determined.

Studies investigating the pathophysiology of ASD have relied on brain imaging, post-mortem tissue analyses, and animal models [9,10]. Although these tools and models have contributed enormously to the study of ASD, they have limitations (i.e., interspecies variation for animal models; individual variability and the limits of the resolution for the available methods of brain imaging; and the “single end-term picture” provided by post-mortem studies, not reflecting the developmental process). In the past decades, however, in vitro models with patient-derived induced pluripotent stem cells (iPSCs) have been developed and shown to consistently reproduce several aspects of human neurodevelopment, providing new opportunities for research in the field of ASD. With the emergence of iPSCs and the subsequent production of brain organoids from these cells, the possibility of manipulating various combinations of morphogenetic processes and the differentiation of lineages involved in organogenesis has increased considerably [11,12]. These structures allow more detailed studies of diverse diseases and disorders, including genotype-phenotype association studies, which contribute to better mimicry of early aspects of brain development and provide important clues for better elucidation of the disorder [13,14]. Therefore, organoids are becoming increasingly relevant for understanding neuropsychiatric disorders, especially more complex ones and those whose origins are not fully understood, such as ASD. In this review, we highlighted the main findings of brain organoid studies aimed at understanding the neurobiology of ASD and the advantages, limitations, and perspectives inherent to the use of this model.

## 2. The Genetics of ASD

ASD is a psychiatric disorder with the highest rate of heritability, presenting a high concordance rate (60–90%) between monozygotic twins and many susceptibility genes [3,15]. Studies based on population data have estimated the heritability of ASD to be 80% or higher [16]. The last few decades have been marked by enormous progress in the ASD genetics field, with several genes and copy number variations (CNVs) identified by microarray and whole exome sequencing studies [17,18]. It is estimated that more than 1000 genes could be involved, with no single gene responsible for more than 1–2% of cases [19,20,21,22,23]. Genetic susceptibility to ASD is highly heterogeneous, with varied combinations of low-risk alleles and rare deleterious variants (found in a lesser proportion in this population).

The heritability of ASD is associated with both common and rare inherited variations, leading to small and large effects, respectively. The identification of rare de novo mutations that interfere with protein function has advanced tremendously over the years. De novo variants can be classified as missense mutations, an acquired modification in which a change in a single nucleotide results in the formation of a different amino acid [21,24], or as nonsense mutations, in which a change in a single nucleotide generates a stop codon that results in truncated protein production [19]. In addition to these alterations, the haploinsufficiency of some genes, such as *CACNA1A*, *SHANK3*, and *SCN2A*, has been linked to ASD [25,26]. In this scenario, only one of the alleles is functional, resulting in an insufficient protein dosage to ensure normal cellular function [27,28,29]. The largest genetic prevalence study targeting ASD analyzed the exome of 11,986 patients with the disorder and revealed that mutations in the *SCN2A*, *SYGAP1*, and *CHD8* genes are most closely related to the development of ASD, but several other genes are also strongly associated with autism [17].

The association between rare or de novo variants with low-risk alleles influences the phenotypic diversity found in patients with rare mutations, with these alleles being responsible for reducing the impact of symptoms on social life [30]. ASD may have a multifactorial cause associated with the genetic etiology of monogenic and polygenic models. The presence of CNVs in the same individual, common single-nucleotide variations (SNVs), or rare SNVs confirms the genetic complexity of autism. However, although genotypic etiologies are diverse, they seem to converge in a limited number of neural pathways and biological events [6].

Among the more robust studies, more expressive CNVs and SNVs were found in genes that encode postsynaptic density proteins and the fragile X mental retardation protein (FMRP) targets [31,32]. Ronemus et al. demonstrated alterations in chromatin-modifying genes and FMRP targets [27]. De Rubeis et al. showed enrichment in proteins involved in neuronal development and axon guidance, signaling pathways, and regulation of chromatin and transcription, as well as increased expression in clusters of proteins involved in growth and transformation pathways of the cell junction and synaptic transmission, in addition to neurodegeneration and transcription regulation [20]. These findings consistently connect ASD risk genes with the functions of excitatory and inhibitory neurons in the developing cortex [6].

ASD-associated variants may affect protein synthesis, transcription, and epigenetic regulation, as well as synaptic signaling, corroborating phenotypically various forms of autism [33]. Several genes were differentially expressed between brain regions, with dysregulation in synaptic function more prevalent in patients with ASD, or even related to immunity and microglial activation, corroborating the hypothesis that abnormal synaptic plasticity and failure in neuronal/synaptic homeostasis could play a key role in ASD susceptibility [34]. The main pathways associated with ASD risk genes are as follows:

1. Chromatin remodeling: mutations in genes that encode regulators of chromatin remodeling and gene transcription that influence neuronal connectivity and synaptic plasticity (i.e., MECP2, *MEF2C*, *HDAC4*, *CHD8*, and *CTNNB1*) [35];
2. Protein synthesis: The levels of synaptic proteins can be influenced by neuronal activity through global and local mechanisms, and in ASD, the regulation of synaptic proteins occurs in an unregulated manner. For example, the mammalian target of rapamycin (mTOR) pathway controls global mRNA translation, and its deregulation may increase the risk of ASD in addition to causing associated diseases, increased cell proliferation, and loss of autophagy. Consequently, suppression of the mTOR pathway, such as *NF1*, *PTEN*, and SynGAP1, causes an increase in translation in neurons and synapses. Mutations in the FMRP complex, *EIF4E*, and *CYFIP1* also increase the risk of autism [36,37];
3. Protein degradation: The ubiquitin-proteasome system, which participates in protein degradation and regulation of synapse composition and is encoded by the UBE3A gene (which encodes ubiquitin ligase), is mutated in patients with Angelman syndrome and is duplicated in the maternal 15q11 chromosome in individuals with ASD [38,39];
4. Synaptic functions: Many proteins encoded by autism risk genes are implicated in neuronal connectivity, glutaminergic pathways (*GRIN2B*), gamma-aminobutyric acid (GABA)ergic pathways (*GABRA3* and *GABRB3*), glycinergic neurotransmission (*GLRA2*), neuritogenesis (*CNTN*), neuronal conduction (*CNTNAP2*), ion permeability (*CACNA1*, *CACNA2D3*, *SCN2A*), synaptic activity (NRXNs and NLGNs), dendritic structure, and neurotransmitter receptors. For example, SHANK deletions, duplications, and coding mutations (*SHANK1*, *SHANK2*, and *SHANK3*) are already well described in patients with ASD; however, they reduce actin accumulation, leading to cellular and dendritic structural dysmorphology as well as axonal outgrowth [40].

Although genomic studies of individuals diagnosed with ASD have increased substantially in recent years, our understanding of the influence of these genetic variants on ASD neurobiology is still insufficient [41,42,43,44]. Animal model studies have not been fully effective, as they are best suited for studies of mutations in a single gene, are time- and cost-consuming, and do not entirely recapitulate the complexity of the human brain [45]. The study of human brain samples is limited by the low availability of post-mortem brain samples from neurotypical people and patients with ASD, and these analyses represent only a static picture of the dynamic processes involved in neurodevelopment [46].

## 3. Brain Organoids

**Figure 1:**
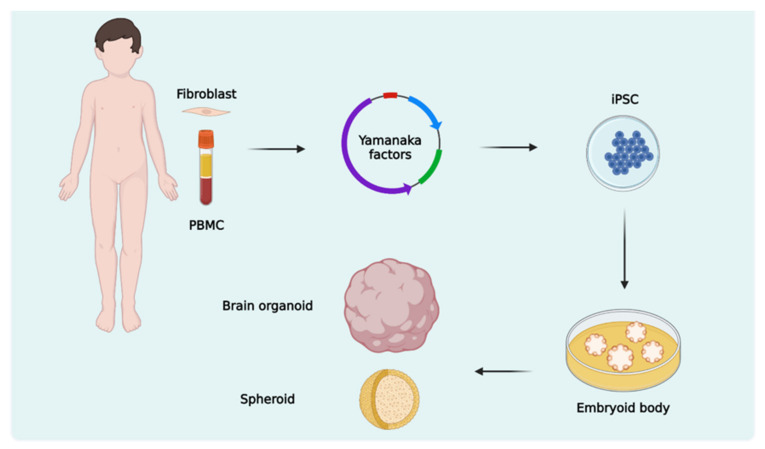
Cell reprogramming and 3D differentiation towards neural tissues. Somatic cells from the patient, such as fibroblasts and peripheral blood mononuclear cells (PBMC), undergo cellular reprogramming from the insertion of Yamanaka factors. These cells become induced pluripotent stem cells (iPSCs) and are used to generate 3D structures such as brain organoids and spheroids.

Using patient-derived stem cells to model neurodevelopmental diseases is not new, as it began with the advent of somatic cell reprogramming technology developed by Yamanaka [12,47]. The iPSCs retain their donors’ genetic background and have been extensively applied in ASD studies through 2D models of differentiation towards neural progenitor cells (NPCs), neurons, astrocytes, and microglia [48]. These studies compared the phenotypes of the differentiated cells between patients with ASD and controls, which can be unrelated or related to neurotypical subjects, or even CRISPR-edited isogenic controls, in which the mutation of interest is introduced or corrected [49]. More recently, brain organoids have emerged as an important model for the analysis of several aspects of the neurobiology of this neuropsychiatric disorder because they improve the in vitro reproduction of the complexity of cellular events and interactions present in early neurodevelopment (Figure 1).

Brain organoids provide a cellular architecture that better recapitulates what is observed during in vivo organogenesis. Therefore, brain organoids are excellent tools to solve some of the gaps left by 2D models and to better reproduce the key features of the developing human brain [50,51]. Interestingly, gene expression and co-expression network analyses have implicated ASD risk genes in the development of excitatory and inhibitory neurons in the inner cortical plate, subventricular zone, and subplate during midgestational, developmental stages, and biological processes that can be modeled with brain organoids [6,52,53].

Organoids are multicellular components that self-organize and develop from stem cells or progenitors to resemble the structure and function of an organ in vivo. In vitro models of the developing brain, such as three-dimensional (3D) models, offer an unprecedented opportunity to study aspects of neuropsychiatric and neurodegenerative diseases at various stages of development [11,54]. Less than ten years have passed since the 1st description of brain organoids, so it is not surprising that ASD studies using organoid models are still not numerous. Nevertheless, organoid research is currently gaining relevance, and one can expect a progressive increase in the number of studies using these structures in the next years [55,56,57].

### 3.1. Protocols and Types of Brain Organoids

The development of 3D models of neural cells began in the 1990s when neurospheres were formed using neuronal progenitor cells [58]. Neurospheres are 3D structures that contain neurons and glial cells but lack the organized cytoarchitecture of the brain tissue [59,60]. A major breakthrough in the development of 3D models occurred a few years after the emergence of iPSCs when Lancaster and colleagues developed the first successful brain organoid protocol [11]. Since then, several researchers worldwide have refined and improved 3D culture protocols directed at the in vitro formation of different specific cortex regions from different types of iPSCs [61,62,63,64,65,66].

Brain organoids can be produced using both unguided and guided methods. Unguided protocols rely on the differentiation and self-organization of cells with minimal induction factors, leading to the formation of cerebral organoids, which are composed of different brain regions and display significant heterogeneity between organoids and batches [11]. In guided methodologies, induction factors are used to direct differentiation towards specific brain regions of interest or regionalized neural organoids [60,67]. Microfilament fibers at the embryoid body (EB) formation stage can refine neuroectoderm formation and enhance cortical development. The cortical region of these organoids presents a better formation of a polarized cortical plate and radial units when compared to unguided brain organoids [68]. Using induction factors, it is also possible to generate forebrain organoids, including the subplate, cortical plate, and Cajal–Retzius cell zones—as well as the three progenitor zones—the ventricular, subventricular, and intermediate zones [69]. After minor adaptations, this model was recently used to analyze the impact of gene mutations on neurodevelopment [70]. In the same methodological context and scenario for evaluating the consequences of gene mutations on neurodevelopment, protocols for developing telencephalic brain organoids have also been developed [61,71].

Small molecules and growth factors have also been used to generate another type of 3D structure: spheroids, which are formed from a single cell type and is less complex than organoids [59]. Spheroids are a good tool for assessing neurotoxic effects when exposed to chemical agents [72]. Furthermore, two or more spheroids representing different brain regions can be fused, thus generating more complex structures called assembloids, making it possible to analyze the interactions between these regions in vitro [60,66,73,74].

**Figure 2:**
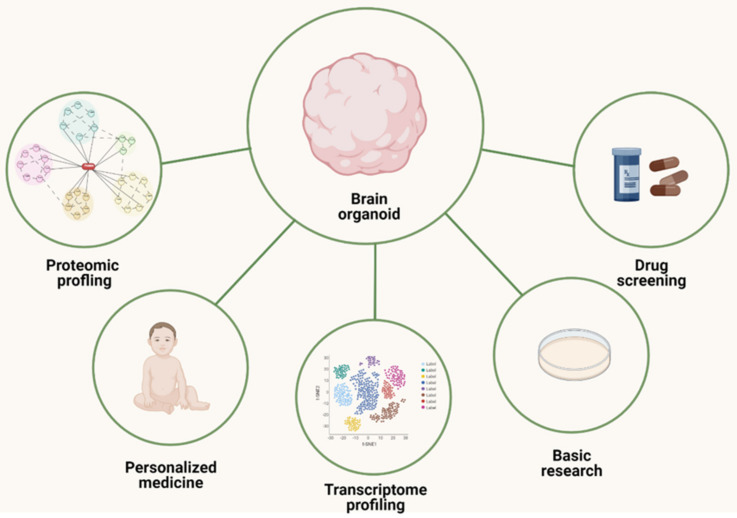
Applications of brain organoids in autism spectrum disorder (ASD) studies. Three-dimensional structures have been used in drug testing to evaluate the protein and gene profile of the cells from which they were generated; they have become an important tool in basic research and enabled the beginning of the emergence of personalized medicine.

Although there have been significant efforts to improve these protocols, the different methods described for obtaining brain organoids or spheroids are complementary. Cerebral organoids may be an ideal model to study gross phenotypes (e.g., microcephaly or macrocephaly or infectious agents such as ZIKV) [11,75,76,77,78]. However, guided protocols provide more consistent and similar organoids with homogeneous morphology, allowing quantitative studies in regions of interest for specific disorders in more detail [50,63]. These structures have been applied to morphological analyses and multi-omics approaches, including bulk proteomics, transcriptomics, and single-cell transcriptomes. With time, brain organoids have increased in complexity and have begun to present a better resemblance to the fetal brain, increasing their utility for studies in the neurodevelopment/ASD field (Figure 2).

### 3.2. ASD Studies with Brain Organoid Models

The application of brain organoids in the study of ASD has led to reports of different phenotypes associated with variations in different genes, influencing transcriptional pathways, morphological features, and the establishment of neuronal networks [3]. The most relevant studies conducted to date that have used brain organoids as a model for understanding the neurobiology of ASD are summarized in Table 1 and are described below. Studies have focused on specific genetic variations, with few studies on the role of environmental factors such as valproic acid exposure.

One of the first studies on Telencephalic organoids produced from ASD patient-derived iPSCs pointed to a link between FOXG1 expression and GABAergic cell development. FOXG1 is one of the transcription factors (TFs) responsible for precursor cell proliferation and differentiation in the brain. In addition, it is a key player in the development of the brain, regulates neocortical neurogenic, and is important to the cortical layer and corpus callosum formation. In this study iPSCs from four families were differentiated into organoids using a modification of the free-floating tridimensional culture method. Transcriptome analysis by bulk RNA-seq, morphological analyses, and the use of RNA interference (RNAi) of the cells originating from the organoids obtained from four idiopathic patients with ASD revealed an association between the overexpression of FOXG1 and the increased production of GABAergic neurons compared to family controls. However, no differences were observed in the production of glutamatergic neurons [61]. Although limited by the small number of patients, these findings support the hypothesis of an excitatory/inhibitory imbalance present in the ASD brain, which originally came from neuropathological observations [79,80].

A recent study analyzed hundreds of brain organoids generated from gene-edited from iPSCs—from ASD patients or CRISPR-edited cell lines—presenting haploinsufficiency of the following ASD risk genes: *SUV420H1* (also known as *KMT5B*), *ARID1B*, and *CHD8* [81]. KMT5B is a Histone-lysine N-methyltransferase involved in histone methylation involved in DNA damage repair and gene silencing. In addition, KMT5B is expressed in the prefrontal cortex, one important brain region that is responsible for regulated executive functions and social cognition [82]. *ARID1B* encodes a subunit of the BRG1/BRM-associated factor (BAF) chromatin remodeling complex and regulates brain development and function [83]. *CDH8* encodes the chromodomain-helicase-DNA-binding protein 8, a transcriptional regulator. As well as *KMT5B* and *ARID1B* genes, *CHD8* is involved with chromatin remodeling and mutations in this type of gene are frequently associated with ASD. To investigate whether mutations in different ASD risk genes converge on shared phenotypes, cortical organoids from different human induced pluripotent stem cell lines were cultivated until 6 months. Among the robust and varied experiments performed, mainly regarding morphological, protein, and gene expression analyses, it was concluded that all the mutations ultimately led to the asynchronous development of two main cortical neuronal lineages, GABAergic neurons and deep-layer excitatory projection neurons, although each one acted through largely distinct molecular pathways. Therefore, these results corroborate the findings of Mariani et al. [61], reinforcing the theory that inhibitory–excitatory imbalance is present in the neurodevelopment of autistic patients and that mutations in distinct genes affecting distinct molecular pathways converge to a common phenotype. This work showed that different mutations in different genes in ASD risk genes in human brain organoids altered distinct molecular pathways that converge to an asynchronous development of shared neuronal classes [81].

*CHD8* has an established role as one of the genes with the strongest association with ASD, and is associated with a unique phenotype that includes the presence of macrocephaly [84]. Cerebral organoids were produced from *CHD8* mutant and control human embryonic stem cell lines (hESCs) to evaluate whether it regulates the expression of other genes involved in brain development implicated in ASD, such as the loss of function of *AUTS2* and *TCF4*. Organoids were analyzed at days 20, 60, and 120, showing differentially expressed genes (DEGs) involved in regulating Wnt/β-catenin signaling, a pathway that has been the target of pharmacological studies. In addition, different populations of GABAergic neurons have been shown to be altered, with unbalanced excitatory/inhibitory neurons [85]. *CHD8* haploinsufficiency was also evaluated in another study on human cerebral organoids. They found that *CHD8* haploinsufficiency disrupted neurodevelopmental trajectories, with an accelerated and delayed generation of inhibitory and excitatory neurons, respectively. This imbalance is consistent with the enlargement of cerebral organoids as an in vitro correlate of patients’ macrocephaly [86].

Considering that ASD has multiple causes, some studies have investigated the contribution of environmental factors to ASD phenotypes. One study employed human iPSC-derived 3D brainorganoids (BrainSpheres) carrying a heterozygote CRISPR/Cas9-introducedinactivat-ing mutation in *CHD8* and exposed to CPF or its oxon-metabolite (CPO) and. The brainspheres were cultivated for 8 weeks and exposed to 100 µM CPF or CPO for 24 h at 4 weeks of differentiation. The results showed that the compounds caused modifications in impaired neurite outgrowth, increased cellular oxidative stress, and an imbalance in neurotransmission. Furthermore, autistic patients with a mutation in this gene may have aggravated symptoms when exposed to certain toxic agents. In addition, the relevance of using the 3D model for studies with risk genes for ASD is associated with environmental factors, such as chemical agents [72].

Another study used forebrain organoids (hFOs) to identify how valproic acid (VPA), an anticonvulsant and mood stabilizer, contributes to ASD risk in humans. Human iPSCs from healthy patients were used to generate hFOs exposed to VPA. The hFOs aged 42 days were exposed to VPA at 1 mM, a clinically relevant concentration for 3 days. Through proteomics, genomics, and electrophysiology analyses, it was found that *AMK4*, *CLCN4*, *DPP10*, *GABRB3*, *KCNB1*, *PRKCB*, *SCN1A*, and *SLC24A2* were affected by VPA. These results significantly overlapped with the pathways found to be dysregulated in the brains or organoids derived from patients with ASD. Single-cell RNA sequencing analysis showed that VPA exposure affected the expression of genes annotated in hFOs in the choroid plexus, excitatory neurons, immature neurons, and medial ganglionic eminence cells. The microelectrode array further identified that VPA exposure of hFOs disrupted synaptic transmission [87].

Another gene associated with ASD is *CNTNAP*, which encodes a member of the neurexin family. The CNTNAP family contains five members from CNTNAP1 to CNTNAP5, and them are involved in the formation of myelin, trafficking potassium channels of the cell membrane, neurite, and synapse development [88]. By analyzing forebrain organoids—cultivated until 26 weeks—derived from patients with ASD carrying the homozygous c.3709DelG mutation in *CNTNAP2*, it was observed that this gene is highly expressed in early-born deep layers/subplate excitatory neurons. Furthermore, an increase in several cortical cells was also observed, which increased the overall organoid volume in patient-derived organoids due to the increased proliferation of neural progenitor cells and other proliferating cells [70]. Using mouse cortical organoids (mCOs) derived from Cntnap2 knockout (KO) mouse induced pluripotent stem cells (miPSCs), researchers observed that the loss of *CNTNAP2* GABAergic neuron defects downregulates the expression of ventral telencephalic progenitor-related transcription factors (TFs) in the ventricular zone, resulting in defects in GABAergic neurons. Drug tests revealed that the antiepileptic drug, retigabine, led to an increase in GABAergic neurons. In this context, it is important to note that brain organoids can also be used to test various drugs that act on the nervous system [89].

Organoids have also been used to study mutations in the d methyl-CpG binding protein 2 gene (*MECP2*) gene. MECP2 is a key epigenetic modulator in the brain, specifically, MeCP2 controls gene expression and modulates chromatin architecture through binding to methylated DNA. The loss-of-function (LoF) of this gene is responsible for Rett syndrome (RTT) [90]. In a recent study, organoids were used to evaluate the therapeutic efficacy of Nefiracetam and PHA 543613, to show whether the drugs can reverse network activity in *MECP2*-KO cortical organoids. The results showed that drugs increased network activity in *MECP2*-KO cortical organoids but did not fully restore activity; therefore, other drugs should be investigated, as well as the gene expression of specific neurotransmitter markers, including cholinergic, GABAergic, and glutamatergic signaling [91]. In a different context, the analyses of the organoids demonstrated increased ventricular area compared to that in the control group, but the mean ventricle wall thickness was decreased, which supports the previous main findings of monolayer neuronal experiments: enhanced proliferation and decreased maturity of these cells [92].

The SHANK family proteins are postsynaptic scaffolding proteins that regulate excitatory synaptic development and function. Specifically, the SHANK3 member is an abundant excitatory postsynaptic scaffolding protein implicated in various neurodevelopmental disorders [93]. *SHANK3* is a recognized ASD-risk gene and was studied by generating telencephalic organoids from isolated self-organized single neural rosettes (SNRs) from a cell lineage with variants in this gene. Organoids were cultivated until 5 months and analyzed at different time points for distinct experiments. Neurons in organoids with a hemizygous deletion of *SHANK3* exhibit intrinsic and excitatory synaptic deficits and impaired expression of several clustered protocadherins [94].

Another study revealed that *Rab39b* deletion promotes PI3K–AKT–mTOR signaling, the inhibition of which rescued enlarged organoid sizes and NPC proliferation caused by *RAB39b* mutations. RAB39B is localized to Golgi vesicles and recycling endosomes and is required for glutamatergic receptor maturation but also for alpha-Synuclein (aSyn) homeostasis and the inhibition of its aggregation [95]. Mutations in the GTPase gene *RAB39b* are associated with ASD and intellectual disability. However, the in vivo roles of RAB30b in the brain are poorly understood. To investigate RAB39b’s functions in human brain development, human pluripotent stem cells and cerebral organoids were used. To delete the human *RAB39b* gene, mutant iPSC lines were generated using the CRISPR/Cas9 approach. These findings corroborate those of other studies presented here, in which changes were observed in organoid morphologies that have gene alterations linked to ASD development, as well as in neuronal populations, when compared with that in control groups [96].

In summary, mutations in genes associated with chromatin remodeling, epigenetic changes, and environmental exposures, in addition to changes in genes associated with migration and formation of neuronal cells have been targets of studies in recent years in order to better understand the neurobiology of ASD. It is notorious that most of the results of the papers presented in this review strongly corroborate one of the main hypotheses linked to ASD: the presence of an inhibitory/excitatory imbalance.

**Table 1:** Studies applying human induced pluripotent stem cell (iPSC)-derived organoids or spheroids to model autism spectrum disorder (ASD).

Ref.	Brain Organoid Type	Aims/Methods	Main Findings
Jong et al., 2021 [70]	Forebrain	Studied the effects of a protein-truncating homozygous mutation in *CNTNAP2* on embryonic cortical development using ASD patients-derived iPSCs	*CNTNAP2* is most highly expressed in several types of excitatory neurons and leads to cortical overgrowth
Lim et al., 2022 [97]	Cerebral	Performed RNA sequencing on samples comprising organoids from CRISPR-edited iPSCs and described a framework (Orgo-Seq) to integrate bulk RNA and single-cell RNA sequence data	*YPEL3*, *KCTD13*, and *INO80E* genes can be associated as driver genes linked with ASD
Mariani et al., 2015 [61]	Telencephalic	Produced organoids from ASD patients-derived iPSCs, recapitulating transcriptional programs present in mid-fetal human cortical development	Overexpression of FOXG1 is associated with the overproduction of GABAergic neurons
Mellios et al., 2018 [92]	Cerebral	Characterize the effects on human neurogenesis and neuronal differentiation brought about by *MeCP2* deficiency using ASD patients-derived iPSCs	Organoids exhibited increased ventricular area and alterations in GABAergic interneuron differentiation
Meng et al., 2022 [87]	Forebrain	Identified mechanisms by which valproic acid (VPA) contributed to ASD risk in humans, using hFOS from healthy donor-derived hiPSC lines;	VPA affected the expression of genes enriched in neural development, synaptic transmission, calcium, and potassium signaling pathways, which have been implicated in ASD
Modafferi et al., 2021 [72]	Brainspheroids	Identified potential synergy between a mutation in *CHD8* and environmental exposure to the pesticide chlorpyrifos, using brain organoids produced from CRISPR-edited iPSCs	Identified metabolic perturbations and disruption of neurotransmitter systems involved in ASD
Paulsen et al., 2022 [81]	Cortical	Analyzed abnormalities that resulted from haploinsufficiency in three ASD risk genes: *SUV420H*, *ARID1B*, and *CHD8* by single-cell RNA sequencing, with both patient-derived and CRISPR-edited iPSCs	Different ASD risk genes converge on a phenotype of asynchronous neuronal development
Pearson et al., 2022 [98]	Cerebral	Analyzed the differential methylation profile of a regulatory region of the *GAD1* gene, using ASD patients-derived iPSCs	In the ASD group, *GAD1* is subject to differential methylation patterns that may indicate variable epigenetic regulation
Trujillo et al., 2021 [91]	Cortical	Evaluated the therapeutic efficacy of two pharmacological compounds in reversing *MECP2*-KO phenotypes, using CRISPR-edited iPSCs	The compounds increased gene expression of specific neurotransmitter markers, such as cholinergic, GABAergic, and glutamatergic signaling, reverting neuropathological phenotypes and networks
Urresti et al., 2021 [99]	Cortical	Investigated the impact of mutations in the 16p11.2 region on neurodevelopmental processes, using ASD patients-derived iPSCs	Dysregulation on neuronal maturation, migration, synaptic processes, and morphology, resulting in defects in neurogenesis
Villa et al., 2022 [86]	Cerebral	Analyzed alterations in *CHD8*, using CRISPR-edited iPSCs	*CHD8* haploinsufficiency disrupted neurodevelopmental trajectories with an accelerated and delayed generation of inhibitory and excitatory neurons, respectively
Wang et al., 2017 [100]	Cerebral	RNA-seq was carried out on *CHD8*+/− and isogenic control (*CHD8*+/+) cerebral organoids, using CRISPR-edited iPSCs	*CHD8* regulates the expression of other genes implicated in ASD: *TCF4* and *AUTS2*
Wang et al., 2022 [94]	Telencephalon	Investigated telencephalic development under normal and autism-associated *SHANK3* deficiency, using CRISPR-edited iPSCs	Neurons in organoids with a hemizygous deletion of the autism gene, *SHANK3*, exhibit intrinsic and excitatory synaptic deficits
Wegscheid et al., 2021 [101]	Cerebral	Defined molecular and cellular causes for the neurodevelopmental abnormalities in patients with *NF1* mutation, using ASD patients-derived iPSCs	Neural stem cell proliferation and neuronal maturation abnormalities were observed and caused by reduced cytokine receptor-like factor 3 (CRLF3) expression and impaired RhoA signaling
Zhang et al., 2020 [96]	Cerebral	Model human neurodevelopmental dysregulation using *RAB39b* mutant cerebral organoids, using CRISPR-edited iPSCs	*RAB39b* mutations result in over-proliferation and differentiation deficits of neural progenitor cells (NPCs)

## 4. Advances, Limitations, and Perspectives

A decade after their first description, brain organoids have contributed significantly to the understanding of the cellular and molecular neurobiology of ASD. Collectively, the main findings show the existence of an imbalance between excitatory and inhibitory pathways in idiopathic ASD. Common pathways that may involve the overexpression of FOXG1, which has been associated with the overproduction of GABAergic neurons, have also been identified. In addition, these models have shed light on the biological role of genes statistically associated with ASD, such as *CHD8*, *SHANK3*, and others. In addition, transcriptome and proteomic studies using brain organoids have revealed that alterations in distinct molecular pathways can lead to similar phenotypes, leading to a better understanding of the consequences of different mutations in the pathophysiology of autism [22,61,81,85].

It is worth noting that a good ability to detect early developmental phenotypes was observed when using brain organoids, which cannot be observed in animal models or human monolayer cultures (such as the aberrant proliferation of progenitor cells); thus, demonstrating the potential that organoids have for modeling human ASD [14]. Another strength is the ability to recapitulate critical stages of neural development in vitro, allowing for the scrutiny of psychiatric disorders that arise at different times during nervous system development, such as autism spectrum disorders [62]. Therefore, it is believed that in the future, there will be in-depth knowledge of the mechanisms present in the diseases, contributing to the personalization of treatment for brain disorders since these models have been more promising in understanding diverse disorders and the human brain when compared to studies with animal models [22]. This allows the development of personalized medicine, in which organoids are directly derived from the affected person’s cells, in addition to testing specific drugs for each individual. However, this will only be possible with the better modeling of these 3D models, making the physiology and tissue development more appropriate [55].

However, despite the many contributions and advantages of these models, there are some limitations to overcome, such as the lack of vascularity, which limits the size of these three-dimensional structures and compromises the late development of certain brain structures [102]. Nevertheless, a recent study made it possible to produce vascularized organoids and observed that they exhibited an increased number of neural progenitors, in line with the possibility that vessels regulate neural development [103]. In addition, researchers are still searching for new standardization methods for culturing brain cells and trying to understand why different batches of neurons, even those cultured from the same donor, may produce different results [104]. Furthermore, a major limitation of brain organoid studies is the low number of patient-derived iPSC lines included in the studies, along with issues regarding the selection of controls.

Another issue that needs improvement is that although the cultures of neurons and organoids form functional synapses, no one has yet managed to maintain these cultures under ideal conditions for the establishment of appropriate circuits because the current 3D organoid protocols seem to favor progenitor cells and do not support the establishment of mature synapses. Therefore, there is still a need to improve mini-brain generation protocols to efficiently support neurophysiological activity, which will be useful in studies on neuropsychiatric diseases [55]. By the year 2022, it will be nine years since the “creation” of brain organoid models [11]. The model organoids have since undergone modifications and improvements to more finely mimic the in vivo environment in the in vitro setting [65].

Owing to the advances in protocols regarding the formation of brain organoids, such as the possibility of vascularization and the possibility of performing drug tests using these structures, which are formed from the patient’s own iPSCs in most cases, a path towards personalized medicine directed to autistic patients is beginning to be created. Finally, it is notable that the use of brain organoids has provided substantial clues about how inhibitory–excitatory imbalance contributes to the neurobiology of autism, as well as making it possible to more reliably identify novel gene and protein markers involved in the disorder.

## Data Availability

Not applicable.

## References

[1] American Psychiatric Association (2022). Diagnostic and Statistical Manual of Mental Disorders: DSM-5-TR.

[2] Maenner M.J., Shaw K.A., Bakian A.V., Bilder D.A., Durkin M.S., Esler A., Furnier S.M., Hallas L., Hall-Lande J., Hudson A. (2021). Prevalence and Characteristics of Autism Spectrum Disorder Among Children Aged 8 Years—Autism and Developmental Disabilities Monitoring Network, 11 Sites, United States, 2018. MMWR Surveill. Summ..

[3] Forsberg S.L., Ilieva M., Maria Michel T. (2018). Epigenetics and cerebral organoids: Promising directions in autism spectrum disorders. Transl. Psychiatry.

[4] Südhof T.C. (2008). Neuroligins and neurexins link synaptic function to cognitive disease. Nature.

[5] Zoghbi H.Y., Bear M.F. (2012). Synaptic dysfunction in neurodevelopmental disorders associated with autism and intellectual disabilities. Cold Spring Harb. Perspect. Biol..

[6] Willsey H.R., Willsey A.J., Wang B., State M.W. (2022). Genomics, convergent neuroscience and progress in understanding autism spectrum disorder. Nat. Rev. Neurosci..

[7] Hulbert S.W., Jiang Y.H. (2017). Cellular and Circuitry Bases of Autism: Lessons Learned from the Temporospatial Manipulation of Autism Genes in the Brain. Neurosci. Bull..

[8] Brant B., Stern T., Shekhidem H.A., Mizrahi L., Rosh I., Stern Y., Ofer P., Asleh A., Umanah G.K.E., Jada R. (2021). IQSEC2 mutation associated with epilepsy, intellectual disability, and autism results in hyperexcitability of patient-derived neurons and deficient synaptic transmission. Mol. Psychiatry.

[9] Wolff J.J., Jacob S., Elison J.T. (2018). The journey to autism: Insights from neuroimaging studies of infants and toddlers. Dev. Psychopathol..

[10] Varghese M., Keshav N., Jacot-Descombes S., Warda T., Wicinski B., Dickstein D.L., Harony-Nicolas H., De Rubeis S., Drapeau E., Buxbaum J.D. (2017). Autism spectrum disorder: Neuropathology and animal models. Acta Neuropathol..

[11] Lancaster M.A., Renner M., Martin C.A., Wenzel D., Bicknell L.S., Hurles M.E., Homfray T., Penninger J.M., Jackson A.P., Knoblich J.A. (2013). Cerebral organoids model human brain development and microcephaly. Nature.

[12] Takahashi K., Tanabe K., Ohnuki M., Narita M., Ichisaka T., Tomoda K., Yamanaka S. (2007). Induction of pluripotent stem cells from adult human fibroblasts by defined factors. Cell.

[13] Ho B.X., Pek N.M.Q., Soh B.S. (2018). Disease Modeling Using 3D Organoids Derived from Human Induced Pluripotent Stem Cells. Int. J. Mol. Sci..

[14] Adegbola A., Bury L.A., Fu C., Zhang M., Wynshaw-Boris A. (2017). Concise Review: Induced Pluripotent Stem Cell Models for Neuropsychiatric Diseases. Stem Cells Transl. Med..

[15] Castelbaum L., Sylvester C.M., Zhang Y., Yu Q., Constantino J.N. (2020). On the Nature of Monozygotic Twin Concordance and Discordance for Autistic Trait Severity: A Quantitative Analysis. Behav. Genet..

[16] Bai D., Yip B.H.K., Windham G.C., Sourander A., Francis R., Yoffe R., Glasson E., Mahjani B., Suominen A., Leonard H. (2019). Association of Genetic and Environmental Factors With Autism in a 5-Country Cohort. JAMA Psychiatry.

[17] Satterstrom F.K., Kosmicki J.A., Wang J., Breen M.S., De Rubeis S., An J.Y., Peng M., Collins R., Grove J., Klei L. (2020). Large-Scale Exome Sequencing Study Implicates Both Developmental and Functional Changes in the Neurobiology of Autism. Cell.

[18] Sebat J., Lakshmi B., Malhotra D., Troge J., Lese-Martin C., Walsh T., Yamrom B., Yoon S., Krasnitz A., Kendall J. (2007). Strong association of de novo copy number mutations with autism. Science.

[19] Sanders S.J., Murtha M.T., Gupta A.R., Murdoch J.D., Raubeson M.J., Willsey A.J., Ercan-Sencicek A.G., DiLullo N.M., Parikshak N.N., Stein J.L. (2012). De novo mutations revealed by whole-exome sequencing are strongly associated with autism. Nature.

[20] De Rubeis S., He X., Goldberg A.P., Poultney C.S., Samocha K., Ercument Cicek A., Kou Y., Liu L., Fromer M., Walker S. (2014). Synaptic, transcriptional and chromatin genes disrupted in autism. Nature.

[21] Iossifov I., O’roak B.J., Sanders S.J., Ronemus M., Krumm N., Levy D., Stessman H.A., Witherspoon K.T., Vives L., Patterson K.E. (2014). The contribution of de novo coding mutations to autism spectrum disorder. Nature.

[22] Wang P., Zhao D., Lachman H.M., Zheng D. (2018). Enriched expression of genes associated with autism spectrum disorders in human inhibitory neurons. Transl. Psychiatry.

[23] Wiśniowiecka-Kowalnik B., Nowakowska B.A. (2019). Genetics and epigenetics of autism spectrum disorder-current evidence in the field. J. Appl. Genet..

[24] Gaugler T., Klei L., Sanders S.J., Bodea C.A., Goldberg A.P., Lee A.B., Mahajan M., Manaa D., Pawitan Y., Reichert J. (2014). Most genetic risk for autism resides with common variation. Nat. Genet..

[25] Trost B., Thiruvahindrapuram B., Chan A.J., Engchuan W., Higginbotham E.J., Howe J.L., Loureiro L.O., Reuter M.S., Roshandel D., Whitney J. (2022). Genomic architecture of autism from comprehensive whole-genome sequence annotation. Cell.

[26] de Almeida Sampaio G.L., Martins G.L.S., Paredes B.D., Nonaka C.K.V., da Silva K.N., Rossi E.A., Dos Santos R.R., Soares M.B.P., de Freitas Souza B.S. (2019). Generation of an induced pluripotent stem cell line from a patient with autism spectrum disorder and SCN2A haploinsufficiency. Stem Cell Res..

[27] Damaj L., Lupien-Meilleur A., Lortie A., Riou É., Ospina L.H., Gagnon L., Vanasse C., Rossignol E. (2015). CACNA1A haploinsufficiency causes cognitive impairment, autism and epileptic encephalopathy with mild cerebellar symptoms. Eur. J. Hum. Genet..

[28] Spratt P.W., Ben-Shalom R., Keeshen C.M., Burke Jr K.J., Clarkson R.L., Sanders S.J., Bender K.J. (2019). The Autism-Associated Gene Scn2a Contributes to Dendritic Excitability and Synaptic Function in the Prefrontal Cortex. Neuron.

[29] Yi F., Danko T., Botelho S.C., Patzke C., Pak C., Wernig M., Südhof T.C. (2016). Autism-associated SHANK3 haploinsufficiency causes *I*_h_ channelopathy in human neurons. Science.

[30] Pugsley K., Scherer S.W., Bellgrove M.A., Hawi Z. (2022). Environmental exposures associated with elevated risk for autism spectrum disorder may augment the burden of deleterious de novo mutations among probands. Mol. Psychiatry.

[31] Pinto D., Delaby E., Merico D., Barbosa M., Merikangas A., Klei L., Thiruvahindrapuram B., Xu X., Ziman R., Wang Z. (2014). Convergence of genes and cellular pathways dysregulated in autism spectrum disorders. Am. J. Hum. Genet..

[32] Ronemus M., Iossifov I., Levy D., Wigler M. (2014). The role of de novo mutations in the genetics of autism spectrum disorders. Nat. Rev. Genet..

[33] Sahin M., Sur M. (2015). Genes, circuits, and precision therapies for autism and related neurodevelopmental disorders. Science.

[34] Auerbach B.D., Osterweil E.K., Bear M.F. (2011). Mutations causing syndromic autism define an axis of synaptic pathophysiology. Nature.

[35] Cohen S., Gabel H.W., Hemberg M., Hutchinson A.N., Sadacca L.A., Ebert D.H., Harmin D.A., Greenberg R.S., Verdine V.K., Zhou Z. (2011). Genome-wide activity-dependent MeCP2 phosphorylation regulates nervous system development and function. Neuron.

[36] Ma X.M., Blenis J. (2009). Molecular mechanisms of mTOR-mediated translational control. Nat. Rev. Mol. Cell Biol..

[37] Budimirovic D.B., Kaufmann W.E. (2011). What can we learn about autism from studying fragile X syndrome?. Dev. Neurosci..

[38] Greer P.L., Hanayama R., Bloodgood B.L., Mardinly A.R., Lipton D.M., Flavell S.W., Kim T.K., Griffith E.C., Waldon Z., Maehr R. (2010). The Angelman Syndrome protein Ube3A regulates synapse development by ubiquitinating arc. Cell.

[39] Mabb A.M., Ehlers M.D. (2010). Ubiquitination in postsynaptic function and plasticity. Annu. Rev. Cell Dev. Biol..

[40] Sassone-Corsi P., Christen Y. (2016). A Time for Metabolism and Hormones.

[41] Velmeshev D., Schirmer L., Jung D., Haeussler M., Perez Y., Mayer S., Bhaduri A., Goyal N., Rowitch D.H., Kriegstein A.R. (2019). Single-cell genomics identifies cell type-specific molecular changes in autism. Science.

[42] Dumas G., Goubran-Botros H., Matondo M., Pagan C., Boulègue C., Chaze T., Chamot-Rooke J., Maronde E., Bourgeron T. (2021). Mass-spectrometry analysis of the human pineal proteome during night and day and in autism. J. Pineal Res..

[43] Khalid M., Raza H., MDriessen T., JLee P., Tejwani L., Sami A., Nawaz M., Mehmood Baig S., Lim J., Kaukab Raja G. (2020). Genetic Risk of Autism Spectrum Disorder in a Pakistani Population. Genes.

[44] Peng J., Zhou Y., Wang K. (2021). Multiplex gene and phenotype network to characterize shared genetic pathways of epilepsy and autism. Sci. Rep..

[45] Howland J.G., Greenshaw A.J., Winship I.R. (2019). Practical Aspects of Animal Models of Psychiatric Disorders. Can. J Psychiatry.

[46] Lee C.T., Bendriem R.M., Wu W.W., Shen R.F. (2017). 3D brain Organoids derived from pluripotent stem cells: Promising experimental models for brain development and neurodegenerative disorders. J. Biomed. Sci..

[47] Takahashi K., Yamanaka S. (2006). Induction of pluripotent stem cells from mouse embryonic and adult fibroblast cultures by defined factors. Cell.

[48] Cheffer A., Flitsch L.J., Krutenko T., Röderer P., Sokhranyaeva L., Iefremova V., Hajo M., Peitz M., Schwarz M.K., Brüstle O. (2020). Human stem cell-based models for studying autism spectrum disorder-related neuronal dysfunction. Mol. Autism.

[49] Pintacuda G., Martín J.M., Eggan K.C. (2021). Mind the translational gap: Using iPS cell models to bridge from genetic discoveries to perturbed pathways and therapeutic targets. Mol. Autism.

[50] Kim J., Sullivan G.J., Park I.H. (2021). How well do brain organoids capture your brain?. iScience.

[51] Eichmüller O.L., Knoblich J.A. (2022). Human cerebral organoids—A new tool for clinical neurology research. Nat. Rev. Neurol..

[52] Willsey A.J., Sanders S.J., Li M., Dong S., Tebbenkamp A.T., Muhle R.A., Reilly S.K., Lin L., Fertuzinhos S., Miller J.A. (2013). Coexpression networks implicate human midfetal deep cortical projection neurons in the pathogenesis of autism. Cell.

[53] Willsey H.R., Exner C.R., Xu Y., Everitt A., Sun N., Wang B., Dea J., Schmunk G., Zaltsman Y., Teerikorpi N. (2021). Parallel in vivo analysis of large-effect autism genes implicates cortical neurogenesis and estrogen in risk and resilience. Neuron.

[54] St Clair D., Johnstone M. (2018). Using mouse transgenic and human stem cell technologies to model genetic mutations associated with schizophrenia and autism. Philos. Trans. R. Soc. B Biol. Sci..

[55] Kelava I., Lancaster M.A. (2016). Stem Cell Models of Human Brain Development. Cell Stem Cell.

[56] Trujillo C.A., Muotri A.R. (2018). Brain Organoids and the Study of Neurodevelopment. Trends Mol. Med..

[57] Ciarpella F., Zamfir R.G., Campanelli A., Ren E., Pedrotti G., Bottani E., Borioli A., Caron D., Di Chio M., Dolci S. (2021). Murine cerebral organoids develop network of functional neurons and hippocampal brain region identity. iScience.

[58] Reynolds B.A., Tetzlaff W., Weiss S. (1992). A multipotent EGF-responsive striatal embryonic progenitor cell produces neurons and astrocytes. J. Neurosci..

[59] Pamies D., Barrera P., Block K., Makri G., Kumar A., Wiersma D., Smirnova L., Zhang C., Bressler J., Christian K.M. (2017). A human brain microphysiological system derived from induced pluripotent stem cells to study neurological diseases and toxicity. ALTEX.

[60] Qian X., Song H., Ming G.L. (2019). Brain organoids: Advances, applications and challenges. Development.

[61] Mariani J., Coppola G., Zhang P., Abyzov A., Provini L., Tomasini L., Amenduni M., Szekely A., Palejev D., Wilson M. (2015). FOXG1-Dependent Dysregulation of GABA/Glutamate Neuron Differentiation in Autism Spectrum Disorders. Cell.

[62] Paşca A.M., Sloan S.A., Clarke L.E., Tian Y., Makinson C.D., Huber N., Kim C.H., Park J.Y., O’rourke N.A., Nguyen K.D. (2015). Functional cortical neurons and astrocytes from human pluripotent stem cells in 3D culture. Nat. Methods.

[63] Yoon S.J., Elahi L.S., Pașca A.M., Marton R.M., Gordon A., Revah O., Miura Y., Walczak E.M., Holdgate G.M., Fan H.C. (2019). Reliability of human cortical organoid generation. Nat. Methods.

[64] Trujillo C.A., Gao R., Negraes P.D., Gu J., Buchanan J., Preissl S., Wang A., Wu W., Haddad G.G., Chaim I.A. (2019). Complex Oscillatory Waves Emerging from Cortical Organoids Model Early Human Brain Network Development. Cell Stem Cell.

[65] Giandomenico S.L., Sutcliffe M., Lancaster M.A. (2021). Generation and long-term culture of advanced cerebral organoids for studying later stages of neural development. Nat. Protoc..

[66] Andersen J., Revah O., Miura Y., Thom N., Amin N.D., Kelley K.W., Singh M., Chen X., Thete M.V., Walczak E.M. (2020). Generation of Functional Human 3D Cortico-Motor Assembloids. Cell.

[67] Pașca S.P., Arlotta P., Bateup H.S., Camp J.G., Cappello S., Gage F.H., Knoblich J.A., Kriegstein A.R., Lancaster M.A., Ming G.L. (2022). A nomenclature consensus for nervous system organoids and assembloids. Nature.

[68] Lancaster M.A., Corsini N.S., Wolfinger S., Gustafson E.H., Phillips A.W., Burkard T.R., Otani T., Livesey F.J., Knoblich J.A. (2017). Guided self-organization and cortical plate formation in human brain organoids. Nat. Biotechnol..

[69] Kadoshima T., Sakaguchi H., Nakano T., Soen M., Ando S., Eiraku M., Sasai Y. (2013). Self-organization of axial polarity, inside-out layer pattern, and species-specific progenitor dynamics in human ES cell-derived neocortex. Proc. Natl. Acad. Sci. USA.

[70] de Jong J.O., Llapashtica C., Genestine M., Strauss K., Provenzano F., Sun Y., Zhu H., Cortese G.P., Brundu F., Brigatti K.W. (2021). Cortical overgrowth in a preclinical forebrain organoid model of CNTNAP2-associated autism spectrum disorder. Nat. Commun..

[71] Mariani J., Simonini M.V., Palejev D., Tomasini L., Coppola G., Szekely A.M., Horvath T.L., Vaccarino F.M. (2012). Modeling human cortical development in vitro using induced pluripotent stem cells. Proc. Natl. Acad. Sci. USA.

[72] Modafferi S., Zhong X., Kleensang A., Murata Y., Fagiani F., Pamies D., Hogberg H.T., Calabrese V., Lachman H., Hartung T. (2021). Gene-Environment Interactions in Developmental Neurotoxicity: A Case Study of Synergy between Chlorpyrifos and CHD8 Knockout in Human BrainSpheres. Environ. Health Perspect..

[73] Workman M.J., Mahe M.M., Trisno S., Poling H.M., Watson C.L., Sundaram N., Chang C.F., Schiesser J., Aubert P., Stanley E.G. (2017). Engineered human pluripotent-stem-cell-derived intestinal tissues with a functional enteric nervous system. Nat. Med..

[74] Birey F., Andersen J., Makinson C.D., Islam S., Wei W., Huber N., Fan H.C., Metzler K.R.C., Panagiotakos G., Thom N. (2017). Assembly of functionally integrated human forebrain spheroids. Nature.

[75] Klaus J., Kanton S., Kyrousi C., Ayo-Martin A.C., Di Giaimo R., Riesenberg S., O’Neill A.C., Camp J.G., Tocco C., Santel M. (2019). Altered neuronal migratory trajectories in human cerebral organoids derived from individuals with neuronal heterotopia. Nat. Med..

[76] Li Y., Muffat J., Omer A., Bosch I., Lancaster M.A., Sur M., Gehrke L., Knoblich J.A., Jaenisch R. (2017). Induction of Expansion and Folding in Human Cerebral Organoids. Cell Stem Cell.

[77] O’Neill A.C., Kyrousi C., Klaus J., Leventer R.J., Kirk E.P., Fry A., Pilz D.T., Morgan T., Jenkins Z.A., Drukker M. (2018). A Primate-Specific Isoform of PLEKHG6 Regulates Neurogenesis and Neuronal Migration. Cell Rep..

[78] Garcez P.P., Loiola E.C., Madeiro da Costa R., Higa L.M., Trindade P., Delvecchio R., Nascimento J.M., Brindeiro R., Tanuri A., Rehen S.K. (2016). Zika virus impairs growth in human neurospheres and brain organoids. Science.

[79] Casanova M.F., Buxhoeveden D., Gomez J. (2003). Disruption in the inhibitory architecture of the cell minicolumn: Implications for autism. Neuroscientist.

[80] Rubenstein J.L. (2011). Annual Research Review: Development of the cerebral cortex: Implications for neurodevelopmental disorders. J. Child Psychol. Psychiatry.

[81] Paulsen B., Velasco S., Kedaigle A.J., Pigoni M., Quadrato G., Deo A.J., Adiconis X., Uzquiano A., Sartore R., Yang S.M. (2022). Autism genes converge on asynchronous development of shared neuron classes. Nature.

[82] Wang Z.J., Rein B., Zhong P., Williams J., Cao Q., Yang F., Zhang F., Ma K., Yan Z. (2021). Autism risk gene KMT5B deficiency in prefrontal cortex induces synaptic dysfunction and social deficits via alterations of DNA repair and gene transcription. Neuropsychopharmacology.

[83] Moffat J.J., Smith A.L., Jung E.M., Ka M., Kim W.Y. (2022). Neurobiology of ARID1B haploinsufficiency related to neurodevelopmental and psychiatric disorders. Mol. Psychiatry.

[84] Weissberg O., Elliott E. (2021). The Mechanisms of CHD8 in Neurodevelopment and Autism Spectrum Disorders. Genes.

[85] Wang H. (2018). Modeling Neurological Diseases With Human Brain Organoids. Front. Synaptic Neurosci..

[86] Villa C.E., Cheroni C., Dotter C.P., López-Tóbon A., Oliveira B., Sacco R., Yahya A.Ç., Morandell J., Gabriele M., Tavakoli M.R. (2022). CHD8 haploinsufficiency links autism to transient alterations in excitatory and inhibitory trajectories. Cell Rep..

[87] Meng Q., Zhang W., Wang X., Jiao C., Xu S., Liu C., Tang B., Chen C. (2022). Human forebrain organoids reveal connections between valproic acid exposure and autism risk. Transl. Psychiatry.

[88] Tong D.L., Chen R.G., Lu Y.L., Li W.K., Zhang Y.F., Lin J.K., He L.J., Dang T., Shan S.F., Xu X.H. (2019). The critical role of ASD-related gene CNTNAP3 in regulating synaptic development and social behavior in mice. Neurobiol. Dis..

[89] Hali S., Kim J., Kwak T.H., Lee H., Shin C.Y., Han D.W. (2020). Modelling monogenic autism spectrum disorder using mouse cortical organoids. Biochem. Biophys. Res. Commun..

[90] Amir R.E., Van den Veyver I.B., Wan M., Tran C.Q., Francke U., Zoghbi H.Y. (1999). Rett syndrome is caused by mutations in X-linked MECP2, encoding methyl-CpG-binding protein 2. Nat. Genet..

[91] Trujillo C.A., Adams J.W., Negraes P.D., Carromeu C., Tejwani L., Acab A., Tsuda B., Thomas C.A., Sodhi N., Fichter K.M. (2021). Pharmacological reversal of synaptic and network pathology in human *MECP*2-KO neurons and cortical organoids. EMBO Mol. Med..

[92] Mellios N., Feldman D.A., Sheridan S.D., Ip J.P., Kwok S., Amoah S.K., Rosen B., Rodriguez B.A., Crawford B., Swaminathan R. (2018). MeCP2-regulated miRNAs control early human neurogenesis through differential effects on ERK and AKT signaling. Mol. Psychiatry.

[93] Yoo T., Yoo Y.E., Kang H., Kim E. (2022). Age, brain region, and gene dosage-differential transcriptomic changes in. Front. Mol. Neurosci..

[94] Wang Y., Chiola S., Yang G., Russell C., Armstrong C.J., Wu Y., Spampanato J., Tarboton P., Ullah H.M., Edgar N.U. (2022). Modeling human telencephalic development and autism-associated SHANK3 deficiency using organoids generated from single neural rosettes. Nat. Commun..

[95] Koss D.J., Bondarevaite O., Adams S., Leite M., Giorgini F., Attems J., Outeiro T.F. (2021). RAB39B is redistributed in dementia with Lewy bodies and is sequestered within aβ plaques and Lewy bodies. Brain Pathol..

[96] Zhang W., Ma L., Yang M., Shao Q., Xu J., Lu Z., Zhao Z., Chen R., Chai Y., Chen J.F. (2020). Cerebral organoid and mouse models reveal a RAB39b-PI3K-mTOR pathway-dependent dysregulation of cortical development leading to macrocephaly/autism phenotypes. Genes Dev..

[97] Lim E.T., Chan Y., Dawes P., Guo X., Erdin S., Tai D.J., Liu S., Reichert J.M., Burns M.J., Chan Y.K. (2022). Orgo-Seq integrates single-cell and bulk transcriptomic data to identify cell type specific-driver genes associated with autism spectrum disorder. Nat. Commun..

[98] Pearson G., Song C., Hohmann S., Prokhorova T., Sheldrick-Michel T.M., Knöpfel T. (2022). DNA Methylation Profiles of *GAD*1 in Human Cerebral Organoids of Autism Indicate Disrupted Epigenetic Regulation during Early Development. Int. J. Mol. Sci..

[99] Urresti J., Zhang P., Moran-Losada P., Yu N.K., Negraes P.D., Trujillo C.A., Antaki D., Amar M., Chau K., Pramod A.B. (2021). Cortical organoids model early brain development disrupted by 16p11.2 copy number variants in autism. Mol. Psychiatry.

[100] Wang P., Mokhtari R., Pedrosa E., Kirschenbaum M., Bayrak C., Zheng D., Lachman H.M. (2017). CRISPR/Cas9-mediated heterozygous knockout of the autism gene CHD8 and characterization of its transcriptional networks in cerebral organoids derived from iPS cells. Mol. Autism.

[101] Wegscheid M.L., Anastasaki C., Hartigan K.A., Cobb O.M., Papke J.B., Traber J.N., Morris S.M., Gutmann D.H. (2021). Patient-derived iPSC-cerebral organoid modeling of the 17q11.2 microdeletion syndrome establishes CRLF3 as a critical regulator of neurogenesis. Cell Rep..

[102] Sun A.X., Ng H.H., Tan E.K. (2018). Translational potential of human brain organoids. Ann. Clin. Transl. Neurol..

[103] Sun X.Y., Ju X.C., Li Y., Zeng P.M., Wu J., Zhou Y.Y., Shen L.B., Dong J., Chen Y.J., Luo Z.G. (2022). Generation of vascularized brain organoids to study neurovascular interactions. eLife.

[104] Shen H., Bocksteins E., Kondrychyn I., Snyders D., Korzh V. (2016). Functional antagonism of voltage-gated K^+^ channel α-subunits in the developing brain ventricular system. Development.

